# Correction: Cyclone Freddy and its impact on maternal health service utilisation: Cross-sectional analysis of data from a national maternal surveillance platform in Malawi

**DOI:** 10.1371/journal.pgph.0007170

**Published:** 2026-08-20

**Authors:** Hussein H. Twabi, James Jafali, Leonard Mndala, Jennifer Riches, Edward J. M. Monk, Deborah Phiri, Regina Makuluni, Luis Gadama, Fannie Kachale, Rosemary Bilesi, Malangizo Mbewe, Andrew Likaka, Chikondi Chapuma, Moses Kumwenda, Bertha Maseko, Chifundo Ndamala, Annie Kuyere, Laura Munthali, Marc Y. R. Henrion, Chisomo Msefula, David Lissauer, Maria Lisa Odland

There are errors in the Funding statement. The correct Funding statement is as follows: This research was funded by the Bill and Melinda Gates Foundation (INV-004839) and, in part, by the Wellcome Trust (206545/Z/17/Z). DL is supported by a National Institute for Health and Care Research (NIHR) Global Health Professorship (NIHR300808) and (NIHR134781), using UK aid from the UK Government to support global health research. The views expressed in this publication are those of the authors and not necessarily those of the NIHR or the UK Government. The funders had no role in study design, data collection, data analysis, data interpretation, writing of the report, or the decision to submit this paper for publication.
