# Supplementary figures and images for: Cyclone Freddy and its impact on maternal health service utilisation: Cross-sectional analysis of data from a national maternal surveillance platform in Malawi
Source: PLOS Glob Public Health. 2024 Aug 28;4(8):e0003565. doi: 10.1371/journal.pgph.0003565 (PMC11356452; doi:10.1371/journal.pgph.0003565)

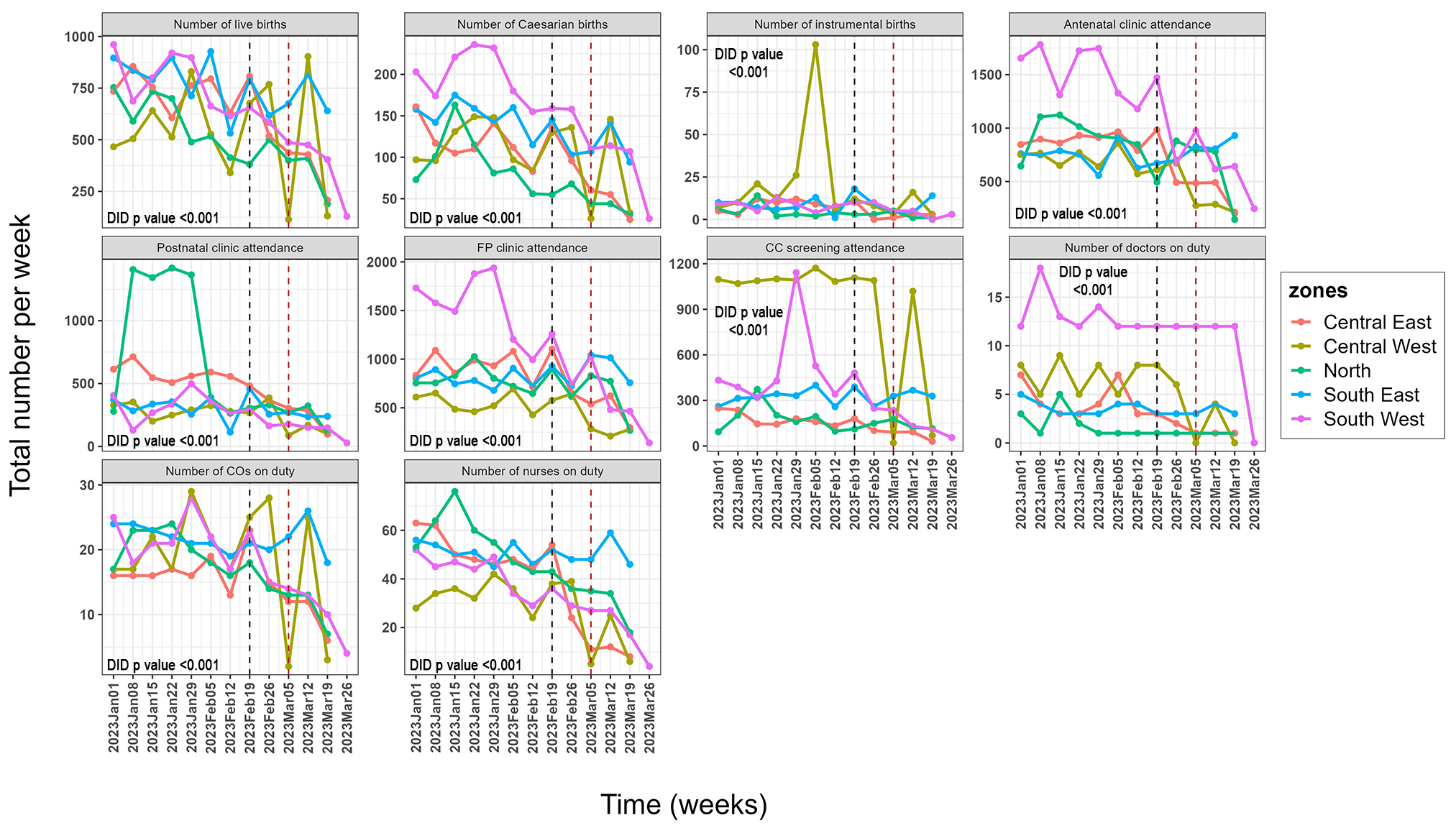

Supplement: S1 Fig — (TIF) [file pgph.0003565.s001.tif]

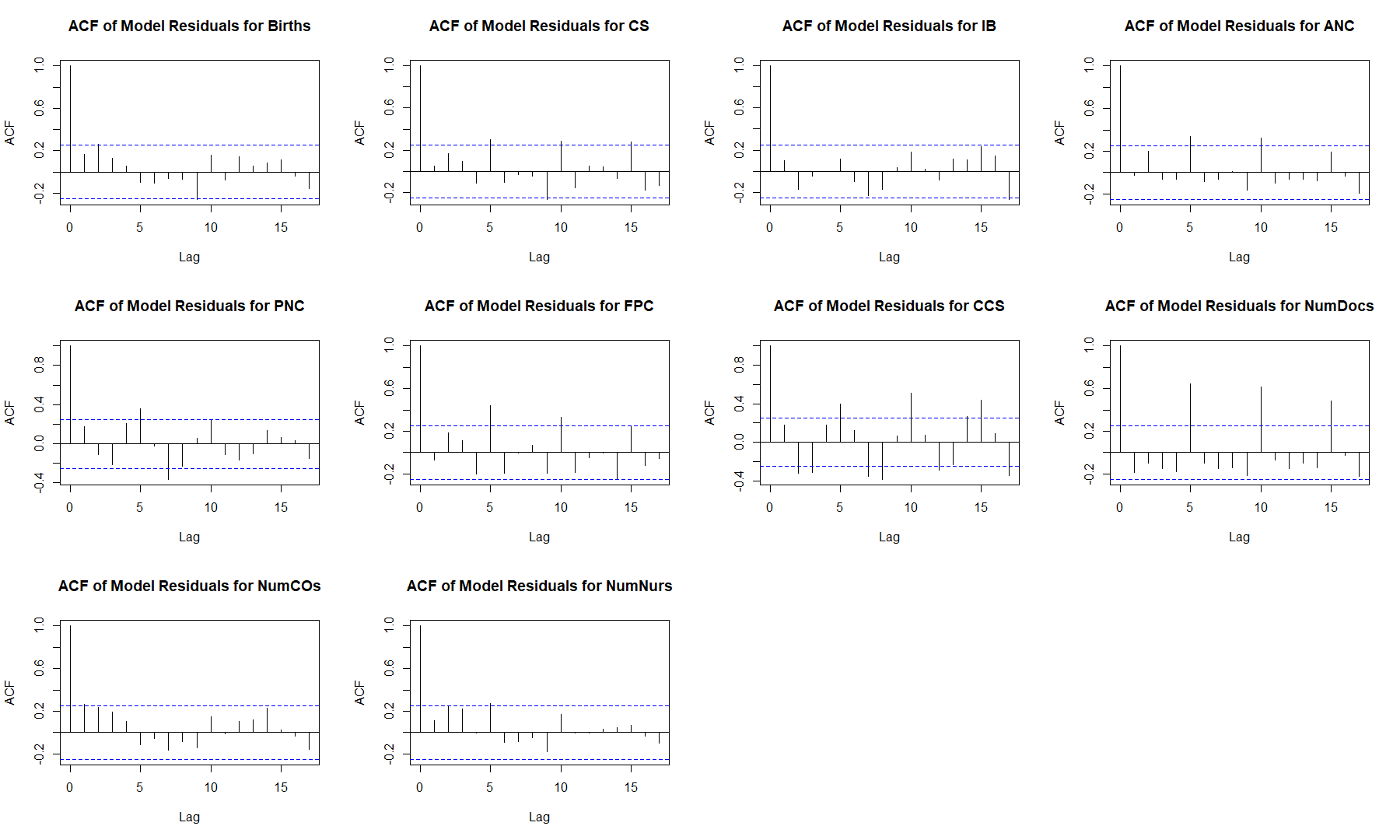

Supplement: S2 Fig — (TIFF) [file pgph.0003565.s002.tiff]

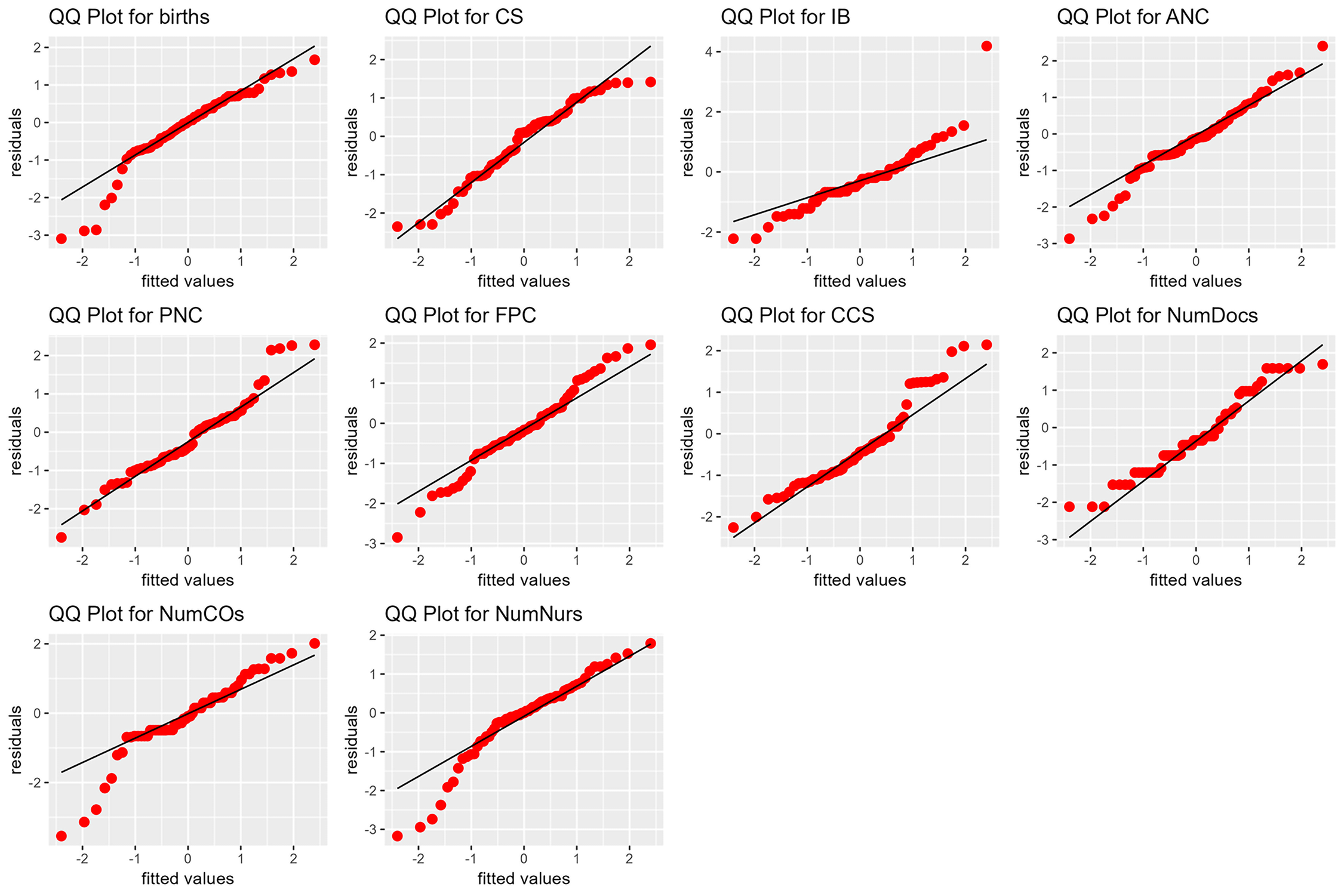

Supplement: S3 Fig — (TIF) [file pgph.0003565.s003.tif]
