# Supplementary material for: Cyclone Freddy and its impact on maternal health service utilisation: Cross-sectional analysis of data from a national maternal surveillance platform in Malawi
Source: PLOS Glob Public Health. 2024 Aug 28;4(8):e0003565. doi: 10.1371/journal.pgph.0003565 (PMC11356452; doi:10.1371/journal.pgph.0003565)
Supplement: S1 Table — (DOCX) [file pgph.0003565.s004.docx]

**Table S1.** Overdispersion testing for fitted Poisson and Negative Binomial Models for trends in uptake of maternal services pre- and post-Cyclone Freddy

| **Variables** | **Poisson Model** | | **Neg Binomial Model** | |
| --- | --- | --- | --- | --- |
|  | **Dispersion ratio** | **P value** | **Dispersion ratio** | **P value** |
| Number of live births | 65.33 | <0.001 | 0.66 | 0.080 |
| Number of Caesarean births | 18.72 | <0.001 | 0.78 | 0.376 |
| Number of instrumental births | 16.42 | <0.001 | 2.79 | 0.000 |
| ANC attendance | 128.84 | <0.001 | 0.88 | 0.672 |
| PNC attendance | 172.81 | <0.001 | 1.48 | 0.136 |
| FPC attendance | 149.94 | <0.001 | 0.98 | 0.952 |
| CCS attendance | 318.85 | <0.001 | 1.19 | 0.488 |
| Number of Doctors | 3.69 | <0.001 | 0.98 | 0.928 |
| Number of COs | 1.90 | <0.001 | 0.85 | 0.400 |
| Number of Nurses | 5.29 | <0.001 | 0.61 | 0.072 |
| *ANC: Antenatal clinic; PNC: Postnatal clinic; FPC: Family Planning Clinic; CCS: Cervical Cancer Screening; CO: Clinical Officer* | | | | |
