# Supplementary material for: Cyclone Freddy and its impact on maternal health service utilisation: Cross-sectional analysis of data from a national maternal surveillance platform in Malawi
Source: PLOS Glob Public Health. 2024 Aug 28;4(8):e0003565. doi: 10.1371/journal.pgph.0003565 (PMC11356452; doi:10.1371/journal.pgph.0003565)
Supplement: Attachment — Submitted filename: response to reviewers.docx [file pgph.0003565.s005.docx]

Dr Hussein H Twabi
Kamuzu University of Health Sciences
Department of Pathology
[husseintwabi@hotmail.com](mailto:husseintwabi@hotmail.com)

11 July 2024

The Editor
PLOS Global Public Health (PGPH)

Dear Editor,

Response to reviewer comments on the manuscript Ref No. PGPH-D-24-00832

Thank you for considering our paper entitled *Cyclone Freddy and its Impact on Maternal Health Service Utilisation: Cross-sectional Analysis of Data from a National Maternal Surveillance Platform in Malawi* for publication in your journal. The comments from the reviewers were graciously received and reflect a depth of knowledge and understanding of the topic at hand. Below are responses to those comments, italicized, after the specific comments by each reviewer.

**Responses to editorial comments**

1. Please send a completed 'Competing Interests' statement, including any COIs declared by your co-authors. If you have no competing interests to declare, please state "The authors have declared that no competing interests exist". Otherwise please declare all competing interests beginning with the statement "I have read the journal's policy and the authors of this manuscript have the following competing interests:"

*Response:* *A conflict of interest statement has been attached to this submission*

2. Tables should not be uploaded as individual files. Please remove these files and include the Tables in your manuscript file as editable, cell-based objects. For more information about how to format tables, see our guidelines: <https://journals.plos.org/globalpublichealth/s/tables>

*Response: The tables have been included in the manuscript document*

3. We have noticed that you have uploaded Supporting Information files, but you have not included a list of legends. Please add a full list of legends for your Supporting Information files after the references list.

*Response: A list of legends for the Supporting Information files has been included after the references list*

4. Please check for typological errors: e.g. RR 29 for the risk ratio of postnatal clinic attendance in the North should be 0.19. (p.3, Findings), "calculated" should be "calculate" and Figure 2A should be Figure 1A (p.6, 3rd paragraph).

*Response: The manuscript has been reviewed and typographical errors corrected*

5. Please check for notation consistency e.g. in how you quote dates on p.4.

*Response: The manuscript has been corrected as suggested*

6. p.5, 4th paragraph: There are seven, not six weeks, from 1st January 2023 to 19th February 2023. You mentioned six weeks (19th February to 30th March 2023) after the cyclone but on p.9 and the first paragraph of the Discussion, you mentioned fix weeks following the cyclone.

*Response: The manuscript has been corrected as suggested*

7. p.6, 2nd paragraph: Would it be more accurate to say "utilisation of maternal and reproductive health services" rather than "access"?

*Response: The paragraph has been corrected as suggested*

8. p.6, negative binomial equation: Please provide some model validation (e.g. checks for temporal correlation in residuals) in the Supplementary Material.

*Response: Additional Supplementary Material has been provided*

9. p.7: Please write the bullet points as text.

*Response: The manuscript has been corrected as suggested*

10. p.8, 3rd paragraph: Could you clarify how the difference-in-difference p-values were calculated? What 2022 and 2023 dates were used to compute the differences in service indicator values?

*Response:* *the Difference-in-Difference (DiD) test was conducted using a Likelihood Ratio Test (LRT). Two models were constructed: The Full model (This modelled the weekly trends in service utilization from 1 January to 30 March with an interaction term between the weekly trends and the year, as well as main effects), and the Reduced model (This model only included the main effects of the weekly trends in service utilisation and the year, but not their interaction). A paragraph has added to page 7 to explain this.*

11. p.10, 2nd paragraph: It would be useful to add the context ("the areas affected by the floods in our study were all but naive to disasters of such magnitude compared to the regions that experience the yearly monsoon floods of Bangladesh") to the Background on p.4.

*Response: Paragraph 3 on page 4 has been edited to provide this context*

12. p.10, 4th paragraph: It is mentioned that "the complex interplay of socio-economic challenges, disrupted infrastructure, and altered community dynamics during and after a natural disaster could decrease healthcare utilisation". Could you comment on this more in the light of the varying impact observed in the different zones (Central East, Central West, North, Southeast and Southwest)?

*Response: The discussion has been expanded to include this*

13. p.11, 2nd paragraph: It is mentioned that the lack of individual-level information prevents the further examination of associations. It may be possible to use zone-level data on e.g. the socio-economic indicators and transport system.

*Response: this may be true, but the dataset provided does not include information about zonal infrastructure, transport systems, and socio-economic indicators. We can make suppositions based on unpublished information, though reliable records are lacking for a comprehensive statistical analysis.*

14. Fig. 1B: Where was this figure referenced in the main paper? What do the colours and numbers in the warning key mean? Is the cyclone past track the actual observed track? When was the forecast track produced?

*Response: The included figure was outdated. The image has been updated, and a reference added in the introduction. The legend has also been updated to provide explanations for all the notations. The colours and the numbers in the risk matrix represent the severity of the risk, combining the likelihood of a disaster occurring with the potential impact of that event*

15. Fig. 3: The first subfigure provides the same information as Table 1. You could either split the figure into two and increase the size of the figure so that the numbers and text are easier to read, or present the rest of the subfigures as tables (similar to Table 1).

*Response: The figure has been edited as per suggestion*

**Responses to Reviewer 1 comments**

1. The background needs to be completed using more valid references and related evidence. The authors need to highlight the problem statement and why they designed this project to address it. what is the added value and the necessity of this research since we know that disasters effects women' s health and I have investigated and reported since years ago. What are the innovations of your research?

*Response: this is formative research that begins to provide background information on how such disasters affect service utilization in Malawi, which is so far lacking. A paragraph has been included to expound on this.*

2. Mention the ethical code

*Response: Paragraph 4 on page 7 has been expanded in response to this.*

3. The discussion needs to be improved by more interpretation of your findings comparing to other similar studies conducted in different settings.

*Response: The discussion has been expanded to address this comment*

4. In the conclusion section, suggest more practical suggestions for your audience and interested stakeholders as well as more suggestions for further research

*Response: The conclusions have been expanded to address this comment*

Responses to Reviewer 2 comments

The manuscript is well written with a good and appealing title. This is an important topic given the current climate changes, emerging and re-emerging diseases, pandemics, disasters and other public health emergencies. It reflects our level of preparedness and readiness to detect, prevent and respond to disasters and other public health emergencies. It provides insight on how to handle essential health services on reproductive, maternal and child health during disaster or public health emergency response.
It is technically sound with data supporting the conclusion. The methodology and ethical considerations are appropriate and rigorous. However, it would have been better to establish whether there were any changes of measured indicators trends for similar comparable time frame in the past 3 or 5 years.

*Response: The data available did not allow for such an analysis*

Sincerely,

Hussein H Twabi

Corresponding author

(On behalf of the study team)
